# Guidance on faecal immunochemical testing (FIT) to help diagnose colorectal cancer among symptomatic patients in primary care

**DOI:** 10.3399/bjgp23X733173

**Published:** 2023-05-26

**Authors:** Nicholas R Jones, Thomas Round, Brian D Nicholson

**Affiliations:** Wellcome Trust doctoral research fellow;; School of Life Course and Population Sciences, and Cancer Prevention Trials Unit, King’s College London.; Cancer Theme Lead, and NIHR Academic Clinical Lecturer.

## INTRODUCTION

The Association of Coloproctology of Great Britain and Ireland (ACPGBI) and the British Society of Gastroenterology (BSG) have published a new guideline around faecal immunochemical testing (FIT) in patients with signs or symptoms of suspected colorectal cancer (CRC).^1^ The guideline was written by a multidisciplinary team including GPs and patient representatives, and includes recommendations for both primary and secondary care. NHS England has subsequently written to all GP practices in England recommending they implement this guideline ‘in full’,^2^ but this has created concern among some GPs regarding a perceived shifting of risk and responsibility to primary care. This article reviews the recommendations made by the ACPGBI/BSG and considers what they mean for primary care clinicians.

## THE NEED FOR IMPROVED PRIMARY CARE DIAGNOSTICS

In the UK, CRC is the fourth most common type of cancer.^3^ Close to 50% of patients are currently diagnosed with stage III or IV cancer.^3^ Symptoms alone have a poor sensitivity for CRC, meaning a high volume of secondary care investigations are required to detect cases if symptom-based criteria alone guide referrals.^1^ Endoscopy services in the UK have been struggling to keep up with referral demands, and waiting times for a colonoscopy lengthened during the COVID-19 pandemic.^4^ It is in this context that the role of FIT has been recently evaluated, to determine whether it can safely triage referrals and better identify high-risk patients than symptoms and non-specific blood tests alone.

## FIT USE IN SYMPTOMATIC PATIENTS SEEN IN PRIMARY CARE

FIT has been shown to be a valuable test for clinicians working in primary care to help triage patients presenting with lower gastrointestinal (GI) symptoms into high-or low-risk CRC groups. For example, the National Institute for Health and Care Excellence (NICE) FIT study, a double-blinded study that included close to 10 000 patients referred for colonoscopy on a suspected CRC pathway, reported that 0.4% of those with a negative FIT below the threshold of ≥10 µg Hb/g faeces were found to have cancer on colonoscopy.^5^ Retrospective cohort studies conducted in primary care have reported a cancer miss rate of 7 cases per 10 000 negative FIT at the same threshold, giving the test a negative predictive value in excess of 99%.^6^ These data demonstrate that a negative FIT can help to identify people at lower risk of CRC. A positive FIT can identify high-risk patients who should be prioritised for urgent investigation. This has been the focus of the recent NHS England letter, which recommends clinicians use FIT as a rule-out test for CRC in primary care, in cases of a FIT result <10 µg Hb/g faeces and no ongoing symptoms of concern.^2^

## SAFETY NETTING FOR NEGATIVE FITS

Despite these reassuring results, clinicians should recognise that the high negative predictive value is because most people with lower GI symptoms in primary care will not have colorectal cancer, so false negative test results will be uncommon irrespective of the accuracy of a test. How well does FIT help identify the minority of patients who do have colorectal cancer? A systematic review of 26 primary care studies reported that the sensitivity of FIT for CRC in symptomatic patients at the threshold of ≥10 µg Hb/g faeces is 87% (95% confidence interval [CI] = 81.0% to 91.6%) with a corresponding specificity of 84.4% (95% CI = 79.4% to 88.3%).^7^ Although these metrics outperform other tests GPs currently use to select patients for suspected cancer referral, such as chest X-ray and cancer antigen 125, it means 1 in 10 patients with CRC will have a negative FIT.

For this reason, the ACPGBI/BSG guideline recommends that patients with persistent lower GI symptoms who have a negative FIT in primary care should still be referred for further investigation, and that local referral pathways should provide an urgent referral route for these patients.^1^ It is important that this recommendation is implemented and that a negative FIT is not presented as a barrier to any subsequent investigation.

Scottish guidelines suggest that clinicians should consider repeating a FIT after 6 weeks in patients with a negative first result who remain symptomatic.^8^ There is some evidence that this might improve sensitivity up to 97%, though with a corresponding reduction in specificity.^1^ The ACPGBI/BSG guideline does not currently recommend repeat use of FIT, citing a need for more research in this area.^1^

## RECTAL BLEEDING AND ANAEMIA

A key change in the guidance is that clinicians are now recommended to use FIT in patients who present with rectal bleeding. This is because FIT detects a breakdown haemoglobin product rather than fresh blood. In the NICE FIT study, the proportion of people with a positive FIT was 26.9% in those with rectal bleeding compared with 15.2% in those without.^9^ The sensitivity and specificity of FIT in people with rectal bleeding was 96.6% (95% CI = 92.2% to 98.9%) and 76.6% (95% CI = 75.0% to 78.1%) respectively.^9^ Patients with rectal bleeding and a negative FIT may still need be referred to secondary care but a flexible sigmoidoscopy is usually a sufficient investigation rather than a full colonoscopy. This is particularly important in patients with unexplained rectal bleeding or persistent symptoms.

NICE suggests that CRC be considered in patients aged ≥50 years with unexplained rectal bleeding or in younger patients who also have abdominal pain, altered bowel habit, weight loss, or iron deficiency anaemia.

The guideline does not make a separate recommendation for FIT use in patients with anaemia. Primary care data demonstrate that FIT remains a helpful triage tool in patients with anaemia with a maintained sensitivity and specificity with lowering haemoglobin.^6^ However, as FIT does not provide any information about the likelihood of malignancy in the upper GI tract or about other causes of anaemia, a negative FIT should not replace a comprehensive work-up of the anaemic patient.

Research is ongoing to establish whether combining blood test findings with FIT could further optimise patient selection for further investigation.

## FIT THRESHOLDS FOR SCREENING

This article has focused on the use of FIT in patients with symptoms or signs of CRC. FIT is also used by the NHS as part of the bowel cancer screening programme, with different thresholds used across the four nations for a positive result ranging from 80 to 150 µg Hb/g faeces. Importantly, all of these thresholds are substantially higher than the current threshold of 10 µg Hb/g faeces used for a positive FIT in symptomatic patients. This means that a recent negative screening test cannot be used to exclude CRC in a patient who later attends with lower GI symptoms.

## DELIVERING FIT IN PRACTICE

If FIT is to be implemented in primary care to inform referrals for suspected CRC, it is of critical important that systems are in place to follow up patients who are provided test kits. Some patients may not return a sample or these may be incorrectly labelled. Recall systems can help to identify these people and ensure that they are offered a review appointment and a repeat or different test can be considered. Laboratories offering FIT must ensure that tests are processed and results reported in a timely manner so that the FIT results do not delay onward investigation of symptomatic patients.

The guideline is also clear that patients should be referred using existing local pathways based on symptoms or other relevant risk factors in circumstances when patients may be unable or unwilling to complete a FIT test. The UK has been an early adopter of FIT to assess for CRC in primary care and is one of the first countries to develop guidelines around its use.^10^ However, these data are applicable to healthcare settings globally.

## CONCLUSION

FIT offers a non-invasive, community-based opportunity to help improve triage of the large number of patients seen in primary care with lower GI symptoms. Patients with a negative FIT, particularly in the context of a normal examination and other investigations, are low risk and may be managed in primary care if symptoms resolve. However, CRC pathways must permit the referral of people with a negative FIT and persistent and concerning symptoms or rectal bleeding for urgent assessment. The take home messages are outlined in Box 1.

**Box 1.** Take-home messages

Up to 1 in 10 people with CRC will have a negative FIT; patients with persistent lower GI symptoms who have a negative FIT in primary care should still be referred for further investigation. FIT detects a breakdown haemoglobin product rather than fresh blood so FIT can be used in patients who present with rectal bleeding. FIT does not provide any information about the likelihood of malignancy in the upper GI tract or about other causes of anaemia. A negative FIT should not replace a comprehensive work-up of the anaemic patient. Bowel screening FIT thresholds are much higher than those for symptomatic patients. A recent negative screening test cannot be used to exclude CRC in a patient who later attends with lower GI symptoms.

*CRC = colorectal cancer. FIT = faecal immunochemical testing. GI = gastrointestinal.*
